# Megalin Expression in Primary Oral Squamous Cell Carcinoma Is Associated with the Presence of Lymph Node Metastases, Vascular Invasion, and Lower Overall Survival

**DOI:** 10.3390/cimb45040180

**Published:** 2023-03-24

**Authors:** Ana Zulijani, Ana Milardović, Zoran Kovač, Berislav Perić, Hrvoje Jakovac

**Affiliations:** 1 Department of Oral Surgery, Clinical Hospital Center Rijeka, 51000 Rijeka, Croatia; 2 Faculty of Dental Medicine, University of Rijeka, 51000 Rijeka, Croatia; 3 Pediatric Intensive Care Unit, Clinical Hospital Center Rijeka, 51000 Rijeka, Croatia; 4 Department of Prosthodontics, Faculty of Dental Medicine, University of Rijeka and Clinical Hospital Center Rijeka, 51000 Rijeka, Croatia; 5 Department of Oral and Maxillofacial Surgery, University Hospital Dubrava, 10000 Zagreb, Croatia; 6 Department of Physiology, Immunology and Pathophysiology, Faculty of Medicine, University of Rijeka, 51000 Rijeka, Croatia

**Keywords:** megalin, oral squamous cell carcinoma, lymph node metastases, vascular invasion, overall survival

## Abstract

Megalin (LRP2) is a rapidly recycling multiligand endocytic receptor primarily expressed in polarized epithelial cells. Although megalin might be involved in tumor growth and invasiveness through several mechanisms, its role has been understudied in the field of molecular oncology so far. The present study aimed to evaluate the impact of megalin expression in oral squamous cell carcinoma (OSCC) on disease progression. Megalin expression was evaluated immunohistochemically in 63 OSCC specimens. Data obtained were retrospectively compared with patient clinicopathological features and their survival. The proportion of megalin-expressing cells in the primary OSCC tissue was significantly associated with metastatic spreading to lymph nodes, vascular invasion and lower overall survival rate. Results obtained by the study suggest that megalin can be considered as a novel molecule involved in OSCC pathogenesis, but also useful as a potential biomarker for cancer progression.

## 1. Introduction

Oral squamous cell carcinoma (OSCC) is the most common locally invasive cancer of the oral cavity [1]. Despite rapidly advancing diagnostics and management, the 5-year survival rate of patients with OSCC is still unsatisfactorily low [2]. A worrying majority of OSCC cases are diagnosed in the advanced stages (stage III-IV) with regional lymph nodes being infiltrated with cancer cells. The infiltrated regional lymph nodes are commonly present in patients with early stage OSCC, but they pose a great diagnostic challenge since they usually remain clinically undetectable. Approximately one third of patients after elective neck dissection show microscopically visible metastases in the cervical lymph nodes [3]. Surgery is the first treatment choice for OSCC, but elective neck dissection is a matter of debate despite a significantly lower recurrence rate [3,4]. Delayed diagnosis of metastatic OSCC usually leads to poor outcomes and/or extensive, mutilating surgical procedures reducing profoundly patient quality of life [5]. Therefore, the current state of affairs urges for research on novel molecules clinically applicable as therapeutic targets, as well as biomarkers of OSCC progression and cancer patient’s prognosis.

Megalin, also known as lipoprotein-receptor-related protein-2 (LRP2) or glycoprotein 330, is a rapidly recycling multiligand endocytic receptor, primarily expressed in polarized epithelial cells. A megalin molecule consists of a large NH2-terminal extracellular domain (4.400 amino acids), a single transmembrane domain (22 amino acids) and a short cytoplasmic tail (213 amino acids) [6,7]. Megalin binds and internalizes a wide range of ligands such as hormones and hormone precursors, vitamin-binding proteins, lipoproteins, enzymes and enzyme inhibitors as well as a variety of carrier proteins [6,7]. When bound by ligands such as metallothioneins and transthyretin, megalin can undergo regulated intramembrane proteolysis (RIP) triggering a signaling pathway resembling the Notch manner, whereby C-terminal soluble megalin intracellular domain (LRP-2-ICD; MICD) is shuttled to the nucleus to directly affect gene expression [8,9,10]. Though the presence of megalin expression may promote tumor growth and invasiveness by two principal ways, by supplying cells with increasingly needed metabolites and by modulating gene expression [11,12,13], megalin has been understudied in the field of molecular oncology so far, and only a few studies emphasized its onco-driving potential [14,15,16,17]. On that trail, Andersen et al. reported that proliferation and survival rates of melanoma cell lines reduced after siRNA-mediated megalin inhibition [17]. Furthermore, megalin gene polymorphisms were associated with prostate cancer recurrence and its progression and patient mortality in one study [14]. Recently, we showed that cancer tissue obtained from OSCC patients gradually overexpresses megalin depending on the tumor cell differentiation [18]. In an effort to expand insights on that topic, in the present study we aimed to assess if clinicopathological features and survival of patients with OSCC are related to megalin expression, assuming it as a possible predictive marker.

## 2. Materials and Methods

### 2.1. Subjects and Tissue Specimens

The study included medical records and archival tissue samples of 63 OSCC patients, of which 39 were males and 24 were females. Patients were being diagnosed and treated at Clinical Hospital Center Rijeka, Croatia, over the period from 2014 to 2019. Formalin-fixed and paraffine-embedded OSCC tissue sections were obtained from the archive of the Clinical Department of Pathology and Cytology, Clinical Hospital Center in Rijeka. Two independent pathologists assessed the histopathological features of specimens and made diagnoses according to the fourth edition of the World Health Organization (WHO) [19] and the eighth edition of the AJCC Cancer Staging Manual [20]. Tissue samples used in the study were obtained from tumors that had originated from the anterior two-third part of the tongue (*n* = 24/63; 38.1%), floor of the oral cavity (*n* = 22/63; 34.9%), buccal mucosa (*n* = 8/63; 12.7%) and alveolar gingiva (*n* = 9/63; 14.3%). All patients included were treated surgically, and those who had undergone neoadjuvant chemotherapy or radiotherapy before the tissue sampling were excluded from the study, as well as those with other oral pathology or systemic disease in their medical history. Regarding the histological grades, 34 cases were well-differentiated carcinomas (grade I), 22 showed moderate cellular differentiation (grade II) and 7 were poorly differentiated (grade III). The patient’s age at the time of diagnosis ranged between 45 and 85 years (median age 65 years). Histopathological parameters assessed in the study included tumor thickness, nodal status and perineural and vascular invasion. Clinical and histopathological features of patients involved in the study, as well as their sociodemographic characteristics, are provided in Table 1. The habit of alcohol consumption was not taken in the analysis due to incomplete anamnestic data. Overall survival was considered the period from the time of diagnosis until the patient died. The patents were being followed up between 7 and 136 months, with a median of 33 months. Healthy oral mucosal tissue was obtained from patients undergoing frenectomy and open corticotomy after they signed informed consent.

### 2.2. Immunohistochemistry

Immunohistochemical staining of megalin protein was conducted using DAKO EnVision + System, Peroxidase (DAB) kit (DAKO Cytomation, Santa Clara, CA, USA) on 4 µm thick serial sections of paraffin-embedded OSCC tissue. In brief, after being deparaffinized and rehydrated, tissues underwent heat-mediated epitope retrieval by microwave heating in a 10-mM citrate buffer of pH = 6.0. Having subsequently been treated with blocking solution, tissue slides were incubated with polyclonal rabbit anti-megalin IgG (H-245, Santa Cruz Biotechnology, Dallas, TX, USA; diluted 1:100 in 1% BSA in PBS) over 12 h in a humid chamber at 4 °C. As a secondary antibody, a peroxidase-labeled polymer linked to goat anti-rabbit antibodies was applied for 30 min at room temperature. Immunoreactions were visualized by 3,3′-Diaminobenzidine (DAB) and sections were counterstained with hematoxylin. Following dehydration, slides were covered with Entellan (Sigma-Aldrich, Hamburg, Germany) and analyzed by an Olympus BX51 microscope equipped with a DP50 camera and Cell^F software (Olympus, Tokyo, Japan). The specificity of antibody binding was verified performing negative controls by substitution of the megalin antibody with an isotype-matched control antibody (polyclonal rabbit IgG, Abcam, Cambridge, UK) applied in the same conditions. Negative control slides did not show immunohistochemical signals.

### 2.3. Immunofluorescence

To assess the relation of megalin expression to the presence of proliferation marker PCNA in tumor tissue, double immunofluorescence staining was carried out on 4μm paraffin-embedded OSCC tissue sections. Briefly, following deparaffinization, rehydration and heat-mediated epitope retrieval, slides were incubated with a blocking solution (1% BSA and 0.001% NaN3 in PBS) for 1 h at room temperature. Immediately afterward, rabbit polyclonal anti-megalin antibody (H-245, Santa 198 Cruz Biotechnology, Dallas, TX, USA; diluted 1:50 in blocking solution) and mouse monoclonal anti-PCNA antibody (Abcam, UK; diluted 1:100 in blocking solution) were added, and slides were hereafter incubated for 12 h at 4 °C in a humid chamber. Tissue sections were then rinsed thrice in PBS being subsequently incubated with Alexa Fluor 488- conjugated donkey anti-rabbit secondary antibody (Thermo Fisher Scientific, Waltham, MA, USA; diluted 1:300 in blocking solution) and Alexa Fluor 555-conjugated goat anti-mouse secondary antibody (Thermo Fisher Scientific, USA; diluted 1:500 in blocking solution) for 1 h at room temperature and in a humid and dark environment. Nuclei were visualized using 4′,6-diamidino-2-phenylindole dihydrochloride (DAPI; Thermo Fisher Scientific, USA; diluted 1:1000 in PBS). Analysis and image capturing were performed using an Olympus BX51 microscope and DP50 camera (Olympus, Japan).

### 2.4. Quantification of Megalin-Positive Cells

One thousand tumor cells per microscopic slide at the center of the tumor were counted of which the number of megalin-positive cells was recorded and the percentage of megalin-expressing cells among the overall number of tumor cells in each slide was calculated. Five randomly selected slides were analyzed per patient and the average value was drawn for each patient. Quantification was conducted by two independent observers.

### 2.5. Statistical Analysis

Statistical analyses were performed by the Statistica software (MedCalc Statistical Software version 14.8.1). Patients’ clinicopathological parameters and percentages of megalin-positive cells were summarized using nonparametric methods. The percentage of megalin-positive cells for each group of patients stratified according to the clinicopathological characteristics was expressed as the median value of the individual patients’ average values. The difference between the central tendencies of the two groups was assessed by the Mann–Whitney test, followed by the Kruskal–Wallis test with post-hoc analysis according to Conover for assessing the difference between several groups. When analyzing survival, the OSCC specimens were considered to overexpress megalin if the percentage of megalin-positive cells exceeded the median value. Survival was analyzed using the Kaplan–Meier method, with the log-rank test for the comparison between the survival curves. *p* values < 0.05 were considered statistically significant.

### 2.6. Ethical Statement

The study was approved by the Ethics Committee of Medical Faculty in Rijeka (protocol code: 003-08/20-01/85, number: 2170-24-09-8-20-3, 1 September 2020) and the Ethics Committee of Clinical Hospital Center in Rijeka (protocol code: 003-05/19-1/121, number: 2170-29-02/1-19-2, 24 September 2019) and was consistent with the ethical guidelines of the Helsinki Declaration.

## 3. Results

**Figure 1:**
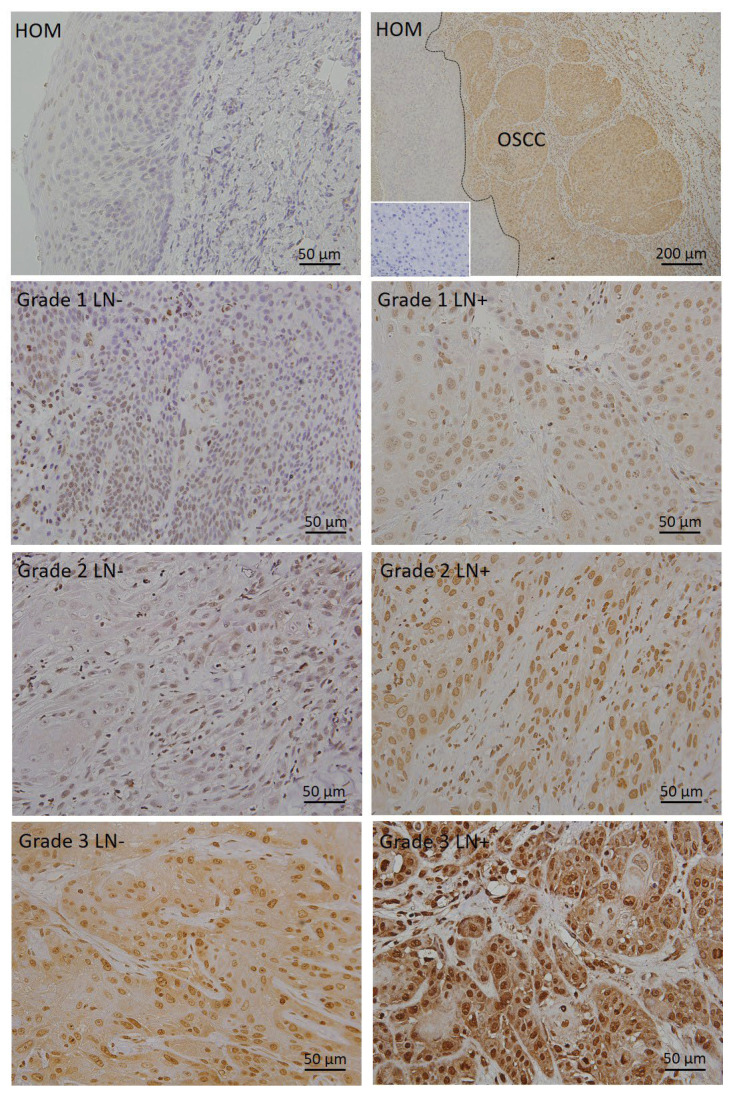
Representative photomicrographs showing megalin immunohistochemical staining in healthy oral mucosa (HOM) and in primary OSCC tissues of different grades (grades 1 to 3), obtained from patients without lymph node metastases (LN−) and patients whose lymph nodes were affected with metastases (LN+) at the time of diagnosis. The inset represents the negative control.

In contrast to the healthy oral mucosa, where we did not detect megalin expression in any case, all 63 OSCC tissue specimens showed megalin immunopositivity. Megalin expression in carcinomas was clearly demarcated from the surrounding healthy tissue (Figure 1). Immunohistochemical positivity was almost completely restricted to the carcinomatous epithelial cells, while the connective tissue within the tumor stroma did not show significant immunohistochemical signals (Figure 1). In the vast majority of positive cells, megalin was localized in the cytoplasm and in the nucleus, indicating its regulated intramembrane proteolysis and nuclear shuttling.

The percentage of megalin-expressing cells significantly increased concomitantly with the loss of carcinomatous tissue differentiation (grade I = 16.9%, grade II = 65.1% and grade III = 94.9%; *p* = 0.000001), in line with our earlier findings based on staining intensity measurement. However, when patients were grouped according to certain clinicopathological characteristics, but regardless of the histological grade of the tumor they had had, we found a significantly higher percentage of megalin-positive cells in OSCC tissue of patients with lymph node metastases when diagnosis had been made compared with the tissue of those whose lymph nodes had not been affected at the time of diagnosis (43.05% vs. 23.9%; *p* = 0.0495) (Table 1, Figure 1).

Similarly, OSCC tissues that showed the presence of invaded microvasculature were found with a significantly higher proportion of megalin-positive cells than tissues with vascular invasion being absent (46.2% vs. 25.8%; *p* = 0.0249) (Table 1). We found no association between the proportion of megalin-positive cells in OSCC tissue and depth of invasion, perineural invasion, TNM staging or sociodemographic characteristics of patients analyzed (Table 1). Furthermore, we did not observe significant differences in the percentage of megalin-positive cells between tumors that originated from different sites in the oral cavity and were obtained from patients with the same clinicopathological characteristics.

**Figure 2:**
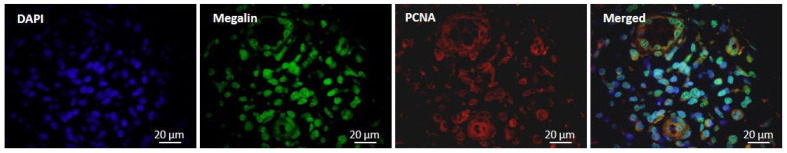
Representative photomicrographs showing double immunofluorescent staining of primary OSCC tissue using anti-megalin and anti-PCNA antibodies.

Seeking to examine whether pro-invasive megalin effects are related to cellular proliferation, we found that proliferation marker PCNA was strictly co-expressed with megalin in all OSCC samples (Figure 2), suggesting enhancement of proliferative capacity as an intermediate factor linking megalin expression with described clinicopathological findings.

**Figure 3:**
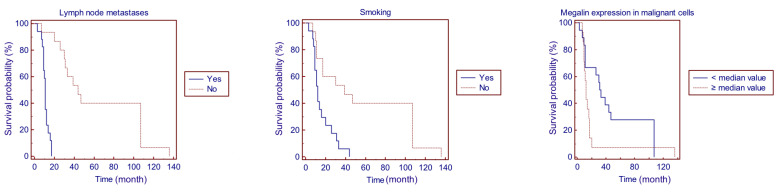
Kaplan–Meier survival curves regarding lymph node metastases, smoking history and megalin expression (<median value/≥median value).

Furthermore, in addition to the well-known conditions and findings reflecting on the life expectancy of OSCC patients, such as smoking habit, lymph node metastases, vascular invasion and TNM stage, using Kaplan–Meier survival analysis we found that megalin overexpression in OSCC tissue (percentage of megalin positive cells ≥ median value) has significantly affected overall survival, whereby patients with megalin overexpressing tumors had significantly lower lifespan (*p* = 0.0462) (Table 2, Figure 3). Perineural invasion, depth of invasion, histopathological grade of tumor as well as age and gender were not associated with the overall survival rate within our patient group (Table 2).

## 4. Discussion

To the best of our knowledge, in the present study, we for the first time demonstrated the association of megalin expression in primary OSCC tissue with the presence of lymph node metastases, vascular invasion and overall survival rate, suggesting that a higher proportion of megalin-expressing cells in tumor specimens could be considered as a possible predictor of poorer prognosis for OSCC patients. Despite the fact that megalin may, owing to its receptor versatility and ability to trigger gene-modulating signaling pathways [11], promote oncogenesis and tumor progression, research on its role in cancer pathobiology has been somewhat neglected so far. Only several studies have linked megalin with malignant transformation, and the literature data offering possible mechanistic insights are almost completely lacking [17,18,21,22]. Pedersen et al. showed acquisition of megalin expression in brain non-Hodgkin lymphoma [22], while Schuetz and colleagues found that clear cell renal cell carcinoma overexpressed megalin [23]. In addition, the study by Andersen et al. demonstrated the induction of megalin in melanoma tissue whereby proliferation potential and survival rates of melanoma cell lines were reduced after siRNA-mediated blockage of megalin synthesis [17]. Decreasing megalin in melanoma cells also resulted in a change in the expression of multiple genes involved in energy metabolism, indicating that megalin contribution to cancer progression could be ensuring energetic competence for rapid proliferation, growth and migration of transformed cells [17]. Since megalin is an archetypical multiligand receptor, support in energy production may also arise from enabling sufficient nutrient uptake into the metabolically highly demanding cancer cells. The mechanism by which megalin affects gene expression is far from clearly understood, but data available indicate that it is mediated by regulated intramembrane proteolysis (RIP) that yields soluble C-terminal megalin intracellular domain (MICD), capable of entering the nucleus, binding DNA and acting as a transcription factor [8,9,10]. OSCC samples of our patients were consistently found with nuclear megalin immunopositivity (Figure 1), strongly suggesting MICD nuclear translocation in the cancer cells. We are obliged to mention here that we used an anti-megalin antibody recognizing epitopes situated at the megalin intracellular domain (H-245, Santa Cruz Biotechnology, USA). Recently, we also demonstrated chromosomal expression of megalin in cancer cells with mitotic figures, which points to the immediate involvement of megalin in the cellular division process [18]. That finding, together with the currently presented co-expression of PCNA and megalin in cancer cells (Figure 2), underlines the pro-proliferative role of the molecule concerned. Furthermore, several growth factors, mitogenic and antiapoptotic molecules, such as surviving [16], clusterin [24], metallothioneins [22] and IGF-1 (insulin-like growth factor-1) [11], have been found to be internalized via megalin. Several studies also pointed out the activation of protein kinase B (PKB; AKT-1) by megalin upon ligand binding, particularly metallothioneins [8,12,13,25,26,27], which is in accordance with our previous results showing colocalization of megalin and phosphorylated AKT in premalignant uterine cervical lesions [21]. Furthermore, Zang et al. showed that megalin overexpression in the NRK-52E cell line led to upregulation of anti-apoptotic factor Bcl-2 while reducing at the same time the expression of pro-apoptotic ones (Bax, Fas, and FasL) [15]. It is interesting and worth mentioning that several in vitro studies found that megalin expression can be autoregulated by a megalin-dependent positive feedback mechanism, whereby MICD, released by RIP and shuttled to the nucleus upon ligand binding, enhances megalin gene transcription [8]. However, there is currently no available data on the presence of this mechanism in cancer tissue.

Data provided by the present study clearly shows the association between megalin expression in primary OSCC tissue and the presence of lymph node metastases at the time of diagnosis, vascular invasion and decreased overall survival rate of patients (Table 1 and Table 2, Figure 3). It should be noted that the presence of lymph node metastases and vascular invasion, being found to be related to megalin expression, was also found as a factor associated with overall survival in an independent analysis, as megalin itself was. However, despite the fact that the TNM staging among other parameters also includes the presence of lymph node metastases and that it affected overall survival (Table 2), we found no association between TNM staging and megalin expression in the group of patients analyzed (Table 1). Such findings could be at least partially explained by principles of the recent guidelines for TNM staging (AJCC 8th Edition of Cancer Staging Manual [20]) which incorporate depth of invasion into the clinical staging of oral cavity carcinoma, and we found no significant association between megalin expression and depth of invasion (Table 1), nor between depth of invasion per se and overall survival rate (Table 2). It is worth mentioning here that there is currently a brisk debate on such staging approach, and recent studies have challenged its prognostic role and relevance, since ambiguity of depth of invasion assessment may undermine its objectiveness [28].

Finally, we have to draw attention to several limitations of our study. The present study included a relatively small number of patients (N = 63), primarily due to strict exclusion criteria that were set to avoid the possible influence of comorbidities. For that reason, we did not have enough samples to perform credible statistical analyses separately for each histopathological grade of the tumor. Though several methods for protein expression analysis are widely used, we considered the immunohistochemical approach the most suitable to be applied on archived tissue, and immunohistochemistry is routinely performed considering the potentially predictive or diagnostic value of our findings. With several existing techniques for quantification of immunohistochemical slides being available (e.g., semiquantitative, staining intensity measurement), the percentage of positive cell calculation was chosen as the most objective and accurate in our opinion. Overall, both the results and limitations of the present study address the need for further research on this topic.

## 5. Conclusions

Data obtained by the study point to megalin expression in primary oral squamous cell carcinoma as being associated with the presence of lymph node metastases, vascular invasion and lower overall survival rate.

## Figures and Tables

**Table 1:** Association between megalin expression in tumor cells and clinicopathological parameters in OSCC patients.

Clinicopathological Characteristic	N	Percentage of Megalin-Positive Cells (Median, Range)	*p*-Value
N = 63			
Gender			
Male	39	40.0 (6.50–99.30)	0.1082
Female	24	24.95 (2.90–96.3)	
Age			
≤65	34	29.40 (5.90–96.60)	0.2495
>65	29	40.00 (2.90–99.30)	
Smoking			
Yes	35	38.60 (7.10–96.30)	0.4849
No	28	28.70 (2.90–99.30)	
Histopathological grade *			
I	34	16.90^1^ (2.90–44.60)	**0.000001**
II	22	65.10^2^ (14.80–85.40)	
III	7	94.90^3^ (70.50–99.30)	
Lymph node metastases			
Yes	28	43.05 (6.50–99.30)	**0.0495**
No	35	23.90 (2.90–96.30)	
Perineural invasion			
Yes	15	38.60 (7.10–85.40)	0.5519
No	43	28.70 (2.90–99.30)	
Vascular invasion			
Yes	15	46.20 (10.60–94.90)	**0.0249**
No	36	25.80 (2.90–89.80)	
Depth of invasion			
≤5 mm	19	19.70 (2.90–99.30)	0.1953
>5 mm	38	39.30 (6.50–89.80)	
TNM *			
I	16	26.85 (2.90–80.10)	0.1853
II	8	18.65 (8.00–96.30)	
III	17	32.90 (6.50–96.60)	
IV	22	41.00 (10.60–99.30)	

* made by Kruskal–Wallis test. The results of the post-hoc analysis are expressed as exponents. All other analyses were performed by Mann–Whitney test.

**Table 2:** Univariate Kaplan–Meier survival analysis with regard to clinicopathological parameters and percentage of megalin-expressing cells in primary OSCC tissue.

Clinicopathological Parameter	Overall Survival (Chi-Squared)	*p*-Value
Gender (male/female)	2.6169	0.1057
Age (<median value/≥median value)	0.9908	0.3195
Smoking (yes/no)	9.8976	**0.0017**
Lymph node metastases (yes/no)	27.4622	**0.0001**
Perineural invasion (yes/no)	1.1767	0.2780
Vascular invasion (yes/no)	13.6816	**0.0011**
Depth of invasion (≤5 mm/>5 mm)	2.8700	0.0902
Histopathological grade (I/II/III)	2.2211	0.3294
TNM (I/II/III/IV)	15.6181	**0.0014**
Percentage of megalin-expressing cells (<median value/≥median value)	3.9729	**0.0462**

## Data Availability

The data presented in this study are available on reasonable request from the corresponding author if data sharing is approved by the ethics committee. The data are not publicly available due to data protection laws and adherence to ethical principles.
